# Three-dimensional assessment of Upper Airway in Class III patients with different facial patterns

**DOI:** 10.4317/jced.60856

**Published:** 2023-10-01

**Authors:** Diego De Nordenflycht, Tomás Corona, Alejandro Figueroa

**Affiliations:** 1 DDS, MSc. Associate Professor, Faculty of Dentistry, Universidad Andres Bello, Viña del Mar, Chile; 2 DDS, Private practice, Viña del Mar, Chile; 3 DDS, Private practice, Quillota, Chile

## Abstract

**Background:**

To evaluate three-dimensionally the upper airway (UA) of class III adults with different facial patterns.

**Material and Methods:**

A cross-sectional study was conducted, in which cone-beam computed tomography (CBCT) images from a private clinic in Viña del Mar, Chile were evaluated. The sample consisted of CBCT images of 59 skeletal class III subjects (33 females and 26 males, mean age 24.7 years) in which the vertical facial pattern was determined using the Vert index, and the minimum cross-sectional area and total volume of the UA were measured. The minimum cross-sectional area variable was analyzed by ANOVA and the total volume was analyzed by Kruskal-Wallis test. Statistical analyses were performed with JASP 0.13.1 software at p=0.05.

**Results:**

The sample included images of 21 brachyfacial, 14 mesofacial and 24 dolichofacial subjects. The mean minimum cross-sectional area of the sample was 591.78 mm2 +/- 149.38 mm2 (minimum=352.00 mm2; maximum=971.00 mm2), being greater in brachyfacial than in dolichofacial and mesofacial subjects, however, these differences were not significant (*p*=0.147). The mean total volume of the sample was 13.40 +/- 4.69 cm3 (minimum=7.16 cm3; maximum=25.66 cm3), being greater in brachyfacial than in dolichofacial and mesofacial subjects, however, these differences were not significant (*p*=0.353).

**Conclusions:**

Considering the limitations of the present study, the vertical facial pattern does not appear to significantly influence upper airway measurements in skeletal class III adults.

** Key words:**Airway, cephalometry, cone-beam computed tomography, facial pattern, malocclusion, Angle class III.

## Introduction

The skeletal classes present structural features that explain the adaptation of the biostructure to the functions of the stomatognathic system such as swallowing, chewing, phonation and breathing (1,2). Regarding the latter, some authors have observed differences between skeletal classes in relation to the size of the airway (3-5). One way to correct skeletal Class III is orthognathic surgery with mandibular setback (6); however, significant changes in the area and volume of the upper airway (UA) have been observed as a result of these surgeries, with potential consequences such as Obstructive Sleep Apnea (OSA) (3,7-9). Therefore, the analysis of the UA is an important aspect of the planning and selection of surgical and orthodontic techniques (3,6-8).

One of the most reported measurements in the study of the UA is the “minimum cross-sectional area”, which corresponds to the area of narrowest diameter between the walls of the UA (7,9). This parameter has been identified as a good predictor of airway changes and risk of OSA, so its evaluation together with the “total volume” of the UA is fundamental in therapeutic planning (3,6). Surgical procedures involving mandibular setback modify the minimum cross-sectional area, increasing the risk of OSA (7), and leading to changes in the apnea-hypopnea index and respiratory event index; a correlation has been observed between the degree of mandibular setback and changes in UA measurements (7), with the oropharynx being the most frequently affected region (6,7,9,10). Kim *et al*. observed a significant decrease in the volume of the UA in Class III patients treated with bimaxillary orthognathic surgery (8).

On the other hand, the vertical facial pattern corresponds to the ensemble of morphological characters that determine the direction of vertical growth of the face, and whose variations are related to different organ systems, such as the orbits, the nasal cavity and the oral apparatus (11). One of the most widely used ways to determine the vertical facial pattern is by calculating the Vert index defined by Ricketts in lateral cephalometry, which classifies subjects as brachyfacial, mesofacial, or dolichofacial (12). The literature on the influence of the facial pattern on the airway is limited (13) and the evidence appears to be inconclusive. Although some previous studies suggested an association between clockwise facial patterns (dolichofacial subjects) and a narrow airway and lower volume (10,13), others reported no significant differences between facial patterns (14).

Given the above, there is a need to study the UA in Class III patients with surgical treatment indications with different facial patterns, in order to estimate the possible impact of the treatment on respiratory function. Therefore, the objective of the present study was to evaluate three-dimensionally the upper airway (UA) of Class III adults with different facial patterns. The tested hypothesis was “there are significant differences in the minimum cross-sectional area and total volume of the UA between different vertical facial patterns in skeletal Class III subjects”.

## Material and Methods

In 2020 a cross-sectional study was carried out in which cone-beam computed tomography images (CBCT) from the database of a private orthodontics clinic (Clínica San Rafael, Viña del Mar, Chile) were evaluated. The study protocol was approved by the scientific committee of the Faculty of Dentistry of Universidad Andrés Bello (Viña del Mar, Chile). Written informed consent was obtained from subjects where images were obtained. All images used in the study were diagnostic images requested by the orthodontic-surgical team for the diagnosis and treatment of each patient, so no subject was unnecessarily exposed to ionizing radiation.

A sample size of 57 subjects was calculated using G*Power software considering an effect size f=0.43, an α-error =0.05, and a power (1-β) =0.80. Among all patients available in the clinic’s records, 70 patients were diagnosed as skeletal Class III using Steiner cephalometry (ANB<0) obtained for evaluation prior to orthodontic-surgical treatment with mandibular setback during the years 2017 to 2020. A sample of 59 images (mean age 24.7 years) who met the inclusion criteria was used. The inclusion criteria were: adults of both sexes, 18 to 35 years old, skeletal Class III with indication of orthodontic-surgical treatment. Exclusion criteria were: history of maxillofacial surgery; presence of temporomandibular disorders; diagnosis of pathology of the upper airway.

CBCT images were taken at 110 kV, 10 mA and 13 seconds of exposure (Planmeca Pro-Max 3D, Helsinki, Finlandia). All CBCT images were taken by the same certified operator. The image capture protocol was the same for all patients: subject standing upright, head in natural position (“looking at the horizon”), teeth in centric occlusion, lips and tongue in resting position, without swallowing, head held with elastic supports. The equipment’s laser guides were used to center the head properly. Each CBCT image contained 512 slices of 1.20 mm, which were saved as DICOM and exported to Planmeca Romexis 5.3.4 software for management and analysis.

The vertical facial pattern was determined in all images using the Vert index with Nemoceph® software version 11.3.0. The Vert index corresponds to the arithmetic mean of the difference between the following cephalometric measurements: facial axis (Ba-Na to Pt-Gn), facial depth (FH to N-Pg), mandibular plane angle (FH to Go-Me), lower facial height (ANS-Xi to Xi-Pm) and mandibular arch (Pm-Xi to Xi-Dc) divided by the standard deviation of those measurements (references values for each measurement varied according to age by the software). Based on these data, subjects were classified as brachyfacial (Vert index >+1.0), dolichofacial (Vert index < -1.0), or mesofacial (Vert index = 0.0) .

**Figure 1:**
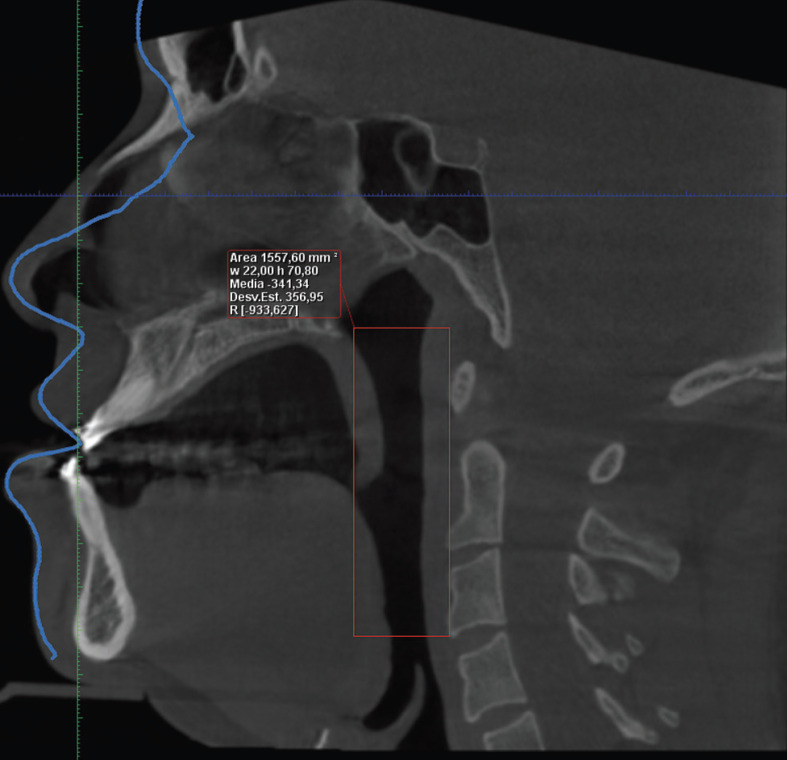
Referential image of CBCT in sagittal view with limits of the UA determined.

**Figure 2:**
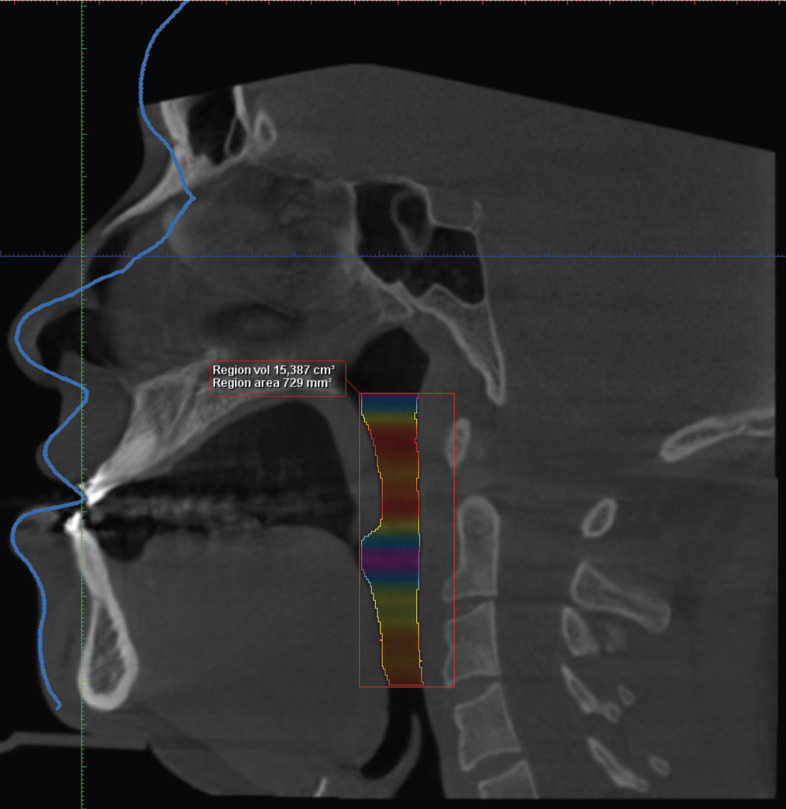
Referential image of CBCT in sagittal view with total volume of the UA recognized by Romexis software.

A three-dimensional analysis of the UA was performed on all subjects’ CBCT images to determine the “minimum cross-sectional area” of the oropharynx and “total volume” of the UA, considering the anatomical limits reported by Guijarro-Martínez and Swennen (15). These measurements was obtained using the Romexis software, which allows a semi-automatic segmentation; in this process, the limits are manually chosen in a grid segment (Fig. 1), and once these limits were determined, the image threshold was adjusted to allow the software to recognize the area occupied by air (darker), and thus calculate the total volume of the UA (Fig. 2). In addition, the narrowest section (or greatest constriction) of the segment was manually determined and the software calculated the minimum cross-sectional area.

Once the measurements of each image had been obtained, the variables “subject sex”, “vertical facial pattern”, “minimum cross-sectional area” and “total volume” were analyzed. The minimum cross-sectional area was defined as the area of the narrowest point of the UA (in mm2). The total volume of the UA was defined as the three-dimensional space (in cm3) of the nasopharynx, oropharynx, and hypopharynx. For each variable, the distribution of data was analyzed using the Shapiro-Wilks test. After the minimum cross-sectional area was analyzed using the Shapiro-Wilks test (*p*=0.067), vertical facial pattern was analyzed by ANOVA and sex was analyzed by t-test. After the total volume of the UA was analyzed using the Shapiro-Wilks test (*p*=0.002), vertical facial pattern was analyzed using the Kruskal-Wallis test, and sex was analyzed using the Wilcoxon Mann-Whitney test. All statistical analyses were performed with JASP 0.13.1 software at *p*=0.05.

## Results

Of the included subjects, 33 were women (55.94%) and 26 men (44.06%). The mean age was 24.62 years (SD=3.57; minimum=19; maximum=33). Regarding the vertical facial pattern, subjects were categorized as follows: 21 brachyfacial (35.59%), 14 mesofacial (23.72%) and 24 dolichofacial (40.67%).

Table 1 shows the descriptive statistics of the minimum cross-sectional area and total volume for each vertical facial pattern. The mean minimum cross-sectional area of the sample was 591.78 +/- 149.38 mm2 (minimum = 352.00 mm2; maximum = 971.00 mm2), being greater in brachyfacial than in dolichofacial and mesofacial subjects. However, the ANOVA showed that these differences between vertical facial patterns were not statistically significant (*p*=0.147). The mean minimum cross-sectional area in males (616.73 +/- 150.52 mm2) was greater than in females (572.12 +/- 147.79 mm2). However, the t-test showed that these differences were not significant (*p*=0.258).

**Table 1:**
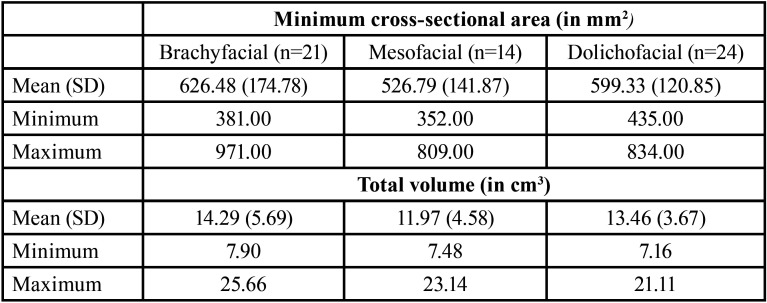
Measurements of minimum cross-sectional area and total volume of UA according to vertical facial pattern.

The mean total volume of the sample was 13.40 +/- 4.69 cm3 (minimum=7.15 cm3; maximum=25.66 cm3), being higher in brachyfacial than in dolichofacial and mesofacial subjects. However, the Kruskall-Wallis test showed that these differences between vertical facial patterns were not significant (*p*=0.353). The mean total volume in males (14.03 +/- 4.38 cm3) was higher than in females (12.91 +/- 4.93 cm3). However, the Wilcoxon Mann-Whitney test showed that these differences were not significant (*p*=0.258).

## Discussion

The present study evaluated the upper airway in CBCT images of skeletal Class III subjects with an indication for orthodontic-surgical treatment, and no significant differences were found between facial patterns in the minimum cross-sectional area and total volume of the UA; similarly, a comparison between sexes also showed that the differences were not significant. Previous studies showed differences in UA morphology in subjects with different skeletal classes, finding that Class II subjects have narrower airways than Class I and III subjects (6). The importance of the present study lies in the fact that in these Class III patients the alternative of surgical treatment with mandibular setback has been identified as a risk factor for the development of obstructive respiratory disorders, such as OSA, since they could reduce the total volume of the UA and the airflow (16).

Several authors have reported studies of UA using conventional lateral cephalometry (9,10,17), which, although a useful method to study the UA in the sagittal plane, provides limited information (6,9,18,19) due to the difficulty of visualizing the axial plane (18,20); therefore, measuring constrictions of the UA in a two-dimensional image does not adequately represent the spatial proximity of structures (18-20). Currently, three-dimensional imaging allows reliable evaluations of the UA (9), and includes techniques such as MRI, endoscopy and computed tomography, among others (3,18,20). CBCT is a low-cost and low-radiation three-dimensional image that reliably differentiates between air, soft tissue, and bone radiographic densities, and its validity for the study of UA has been demonstrated (3,9,20).

Evaluation of the size, shape and volume of the UA begins by defining the volume corresponding to the airway passage in a process called segmentation, which is defined as the construction of 3D virtual surface models (called segmentations) to match the volumetric data (21), in which specific elements are separated and all the unnecessary structures that are not part of the analysis are removed (19). Segmentation can be done manually by the operator, however, it is time consuming and almost impractical for clinical applications (19); on the other hand, semi-automatic segmentation is a significantly faster approach in which the software differentiates the air from the structures by using the differences in density values (19), which is why it was the method chosen in this study. Aside from the method, the literature is inconclusive regarding a standard definition of the UA segments (15,22,23). Various studies have used different anatomic limits, making comparison between them difficult (3,4,6,8,13). The Guijarro-Martínez and Swennen criteria were used in the present study; these were developed for use in three-dimensional imaging to delimit the segments of the UA (15). Another factor that could influence the result of the UA measurements is age; major changes in the UA have been reported to occur from adolescence to adulthood (24). To eliminate the potential influence of age in the present study, only postpubertal subjects were selected, as the average age of the sample being 24.62 years.

Comparison of the UA by sex revealed that the sample contained a similar number of male and female subjects (45% and 55% respectively), and that males had a higher minimum cross-sectional area and total volume of the UA compared to females which can be attributed to sexual dimorphism; however, these differences were not significant (*p*=0.258). This result is consistent with previous reports (3,13,25,26), although other studies showed significant differences between male and female subjects (14,27,28). These differences between studies could be due to the characteristics of the studied sample, e.g. stage of development of the airway, the length of which increases up to the age of 15 and then stalls without further changes in female subjects but continues to increase in male subjects (28). The present study selected adults between 19 and 33 years in whom it has been estimated that the UA is completely developed (24). On the other hand, Grauer *et al*. observed that the UA of male subjects presented a significantly greater volume;however they reported a segmentation process different to the one used in the present study, which could explain the differences (14).

The absence of significant differences between sexes is not consistent with the probability of suffering breathing disorders, moreover the evidence shows a greater risk of OSA in male subjects (26,27). These differences could be explained by anatomical differences, since, despite having similar dimensions of the UA, the air column is surrounded by physical structures of greater mass and volume in the neck of males that require a greater effort to maintain permeability (26). Therefore, the presence of anthropometric differences in the UA between males and females may be relevant in planning treatment with mandibular setback in Class III men due to the increased risk of sleep-disordered breathing and UA collapse (27).

Regarding the comparison of the UA between different vertical facial patterns, no statistically significant differences were observed in the minimum cross-sectional area between facial patterns; however, it was greater in brachyfacial than in dolichofacial and mesofacial subjects. It has been reported that a greater angle of the vertical growth pattern influences the dimensions of the UA, decreasing its volume in mesofacial and dolichofacial subjects (10,29). Flores-Blancas *et al*. observed that brachyfacial subjects have larger UA sizes in the nasopharynx, without significant differences in the oropharynx (17), similarly to previous reports (17,30). On the other hand, other studies have postulated that vertical facial patterns influence the dimensions of the UA (10,29), which could be due to the different characteristics of the samples studied (e.g. different skeletal Class), which makes it difficult to compare results. An important aspect to point out is that both previously cited studies measured the UA on two-dimensional images, so any comparison with studies that use three-dimensional images is complex.

Similar to the minimum cross-sectional area, brachyfacial subjects had a greater total UA volume than dolichofacial and mesofacial subjects, but the differences were not significant (*p*=0.353). Previous studies have observed significant differences in the volume of the UA between subjects with different vertical facial patterns, where patients with a low angle (associated with brachyfacial subjects) had a greater volume than patients with a normal (mesofacial) or higher (dolichofacial) angle (13). The difference in mean UA volume between vertical facial patterns could be due to the methodology for determining the vertical facial pattern, and the skeletal Class of the study subjects, among other factors. Regarding the latter, Grauer *et al*. observed significant differences in UA volume between subjects of different skeletal classes, however, no significant differences were found between subjects with different vertical facial patterns (14). Future studies should evaluate the UA of Class III subjects with different vertical facial pattern after mandibular setback surgery to compare them with the present and the literature findings.

Finally, the present study has limitations that must be considered when interpreting its results: (i) a larger probabilistic sample could increase the statistical power and strengthen the method; (ii) although the focus of study was CBCT images, a more precise evaluation of the vertical facial pattern and the characteristics of the UA of each subject would require clinical information; (iii) although tongue position was standardized as “resting position, without swallowing”, it has been stated that tongue position could be an important parameter when determining UA volume, but recent evidence indicates that standardized method for head and tongue positioning during three-dimensional image acquisitions is lacking and natural head position is suggest for CBCT acquisition (31); (iv) in addition to skeletal Class and facial pattern, other anthropometric characteristics, not considered as study variables, can significantly influence the dimensions of the UA, such as the presence of obesity (32).

## Conclusions

Taking into account the limitations of the study, the results suggest that sex and vertical facial pattern do not significantly influence the volume of the UA, nor are they factors that determine a significant narrowing of the UA in skeletal Class III subjects.
